# Serum Folic Acid and Erectile Dysfunction: A Systematic Review and Meta-Analysis

**DOI:** 10.1016/j.esxm.2021.100356

**Published:** 2021-05-26

**Authors:** Yuyang Zhang, Wei Zhang, Yutian Dai, Hui Jiang, Xiansheng Zhang

**Affiliations:** 1 Department of Urology, The First Affiliated Hospital of Anhui Medical University, Anhui Province, China; 2 Institute of Urology, The First Affiliated Hospital of Anhui Medical University, Anhui Province, China; 3 Anhui Province Key Laboratory of Genitourinary Diseases, Anhui Medical University, Anhui Province, China; 4 Department of Andrology, Drum Tower Hospital, Medical School of Nanjing University, Nanjing, China; 5 The Department of Urology, Peking University Third Hospital, Beijing, China

**Keywords:** Folic Acid, Erectile Dysfunction, Meta-Analysis

## Abstract

**Introduction:**

The association between folic acid (FA) and erectile dysfunction (ED) was contradictory in the published original articles, and no meta-analysis was conducted to pool these data.

**Aim:**

To verify the role of FA in the pathology of ED and explore the treatment efficacy of FA for ED patients.

**Methods:**

An extensive search was performed on PubMed, Cochrane Library, and Web of Science to obtain all relevant studies published up to October 31, 2020. Studies comparing the serum FA level between ED patients and healthy controls, or comparing the score of the IIEF-5, or IIEF before and after folic acid therapy alone or combination in ED patient were eligible for our meta-analysis. The Newcastle-Ottawa Scales (NOS) was used to qualify included studies.

**Main Outcome Measures:**

The standardized mean differences (SMD) and their corresponding 95% confidence intervals (95% CIs) were calculated to pool our data.

**Results:**

Nine studies were eligible for our meta-analysis to verify the association between FA and ED, and to explore the treatment efficacy of FA for ED patients. The pooled SMD of the FA level difference between ED patients and healthy subjects was -0.94 (95% CI: -1.59, -0.30, *P* = .004). Moreover, the level of folic acid in healthy subjects, Mild ED patients, Moderate ED patients and Severe ED patients was 11.847 (95%CI = 9.671, 14.022), 9.496 (95%CI = 8.425, 10.567), 6.597 (95%CI = 5.187, 8.007) and 5.623 (95%CI = 3.535, 7.711) respectively. The SMD of changes in score of IIEF-5 was 1.89 with 95%CI (1.60, 2.17) after FA administration in ED patients. Our analysis also showed that combination therapy of FA plus tadalafil changed the score of IIEF with 0.90 (95%CI = 0.44, 1.36) comparing to combination of placebo plus tadalafil.

**Conclusion:**

This novel meta-analysis demonstrated that FA was an independent risk factor for ED and FA supplement may have potentially positive effects in the treatment of ED patients.

**Zhang Y, Zhang W, Dai Y, et al. Serum Folic Acid and Erectile Dysfunction: A Systematic Review and Meta-Analysis. Sex Med 2021;9:100356.**

## INTRODUCTION

Erectile dysfunction is defined as the inability to reach or maintain an erection that is sufficient for sexual performance.^1^ Among the general population, 2-40% of men aged 40-69 years are affected by this male sexual dysfunction.^2^ The prevalence of ED will reach to 322 million men in 2025.^3^ It has a significant effect on the quality of life of the patients and their partners.^4^ Vascular ED is the most important and prevalent subtype of ED owing to the vascular network of the penis.^5^ The risk factors of vascular ED mainly include obesity, diabetes mellitus (DM), hypertension (HT), dyslipidemia, metabolic syndrome (MetS), lack of exercise, and smoking.^6^ Endothelial dysfunction (EnD) has been found to be a key bridge linking these risk factors and vascular ED.^7^

Nitric oxide (NO), released from the endothelial cell had an important role in initiating and maintaining the erection process.^8^ It mediates the penile erection through the cyclic guanosine monophosphate (cGMP) pathway.^9^ Endothelial cell owns the nitric oxide synthase (eNOS) responsible for formation of NO. So when EnD occurs, it will lead to the reduction of the expression and activity of the eNOS and result in reduced synthesis of NO.^10^ On the contrary, uncoupling of the eNOS could result in the reduction of NO in the endothelial tissue and thus causes EnD.^11^

Folic acid (FA), is found to have important role in the metabolism of NO by initially inverting NOS uncoupling.^12^^,^^13^ It has proved the level of FA was related to EnD.^14^ So, several studies concentrate on the relationship between serum level of FA and ED considering the relation between EnD and ED. To verify the role of FA in the pathology of ED, two type of clinical researches were conducted including comparing the level of FA between ED and healthy subjects and comparing the score of the briefed International Index of Erectile Function (IIEF-5) before and after the FA administration. But totally contradictory conclusions were drawn by the authors. Many studies have shown that patients with ED had lower level of FA comparing to the healthy subjects. However, several studies didn't support this opinion. These studies enrolled small sample, making the conclusion less convincing. Therefore, we conduct this systematic review and meta‐analysis to provide more solid evidence by synthesizing the limited data. To our knowledge, this is the first systematic review and meta‐analysis to explore the role of FA in the pathology of ED. Two type of meta-analyses are included in our meta-analysis: 1 comparing the serum FA between ED patients and healthy subjects, and the other comparing the IIEF-5 changes before and after FA administration.

There were different conclusions on the role of FA on ED etiology, and less was known to the treatment efficacy of FA on ED patients. We perform this meta-analysis to investigate the questions: If serum FA level was different between ED patients and healthy subjects and it decreased significantly with the severity of ED, If FA had potential positive treatment effects for ED patients?

## MATERIAS AND METHODS

We performed this systematic review and meta‐analysis under the guidelines of the Preferred Reporting Items for Systematic Reviews and Meta-Analyses (PRISMA) Statement.^15^ This meta-analysis was registered in the International Prospective Register of Systematic Reviews (PROSPERO) with the ID CRD42020220761. No ethic problems were involved in our meta-analysis, no approval from the Medical Ethics Committee was needed.

### Literature Search Strategies

An extensive search was performed on PubMed, Cochrane Library, and Web of Science to obtain all relevant studies published up to October 31, 2020 without any restrictions. We performed the search using the combination of the following Medical Subject Headings (MeSH) terms: folic acid (MeSH) and erectile dysfunction (MeSH). The search strategies were adjusted according to the demands of the used databases. To obtain extra publications, we screened all references of the potentially relevant studies meeting the pre-set inclusion criteria. The literature searching process was conducted by two authors (Yuyang Zhang, Wei Zhang) independently to avoid missing useful publications.

### Inclusion and Exclusion Criteria

All studies could be enrolled if they comprised all the following criteria: 1) reporting subjects older than 18 years old; 2) comparing the serum folic acid level between ED patients and healthy controls, or comparing the score of the IIEF-5 before and after folic acid therapy in ED patients; 3) reporting quantitative data for the related outcomes; 4) using a validated instrument for the diagnosis of ED. Studies were excluded if they didn't show related outcomes with quantitative data. Of course, reviews, animal experiments, case reports, comments, editorials letters and congress were all excluded.

### Data Extraction and Quality Assessment

Two authors assessed quality and extracted the data of the included studies using the predesigned form independently. A consensus was hold to resolve the conflicts raised by the two authors.

As for the studies evaluating the folic acid level between the ED patients and healthy subjects, the following data were extracted from each included study: first author, study year, study country, definition of ED, number of ED patients/healthy subjects, the folic acid level between the ED patients and healthy subjects. If the study was related to comparing the score of the IIEF-5 before and after folic acid therapy in ED patients, the data were extracted from the original study including, first author, study year, follow-up months, doses of the FA, number of the study and the IIEF-5 before and after the FA administration.

The quality of the case-control study and the cohort study were assessed using the Newcastle-Ottawa Scales (NOS).^16^ Studies were scored through the following categories, selection process, comparability of cohorts and the outcomes ascertainment.

Studies of moderate to high quality was defined with the NOS score 5-9 in our meta-analysis.

### Outcome Measures and Statistical Analysis

The first outcome of interest in our meta-analysis was to compare the folic acid level between the ED patients and healthy subjects. The standardized mean differences (SMD) and their corresponding 95% confidence intervals (95% CIs) were calculated to evaluate the relationship. The second outcome of interest in our meta-analysis was to compare the score of the IIEF-5 before and after folic acid therapy in ED patients. And the results were showed using the same data indicators.

Data analysis was performed with Stata software (version 16.0; Stata Corporation, College Station, TX, USA) and Open Meta-Analyst (completely open-source, funding from AHRQ, grant number: R01HS018574). The statistical significance of the pooled results existed when the P value < 0.05 of the Z tests. When synthesizes the data of the included studies, the Q test or I^2^ value were calculated for evaluating the heterogeneity. Fixed-effect model or random-effect model was chose owing to the heterogeneity. If the I^2^ <50% or p >0.05, we used the fixed-effect model considering no obvious heterogeneity among the studies. Otherwise, the random-effect model was used considering the high heterogeneity. Subgroup analysis or sensitivity analysis were performed when the heterogeneity existed to find the source of it. Each study was eliminated once at a time to assess the influence of the eliminated study to the pooled results. In order to detect the bias of publication, the Begg's test were used. There were bias of publication when the P value <0.05.

## RESULTS

### Study Search and Characteristics

**Figure 1:**
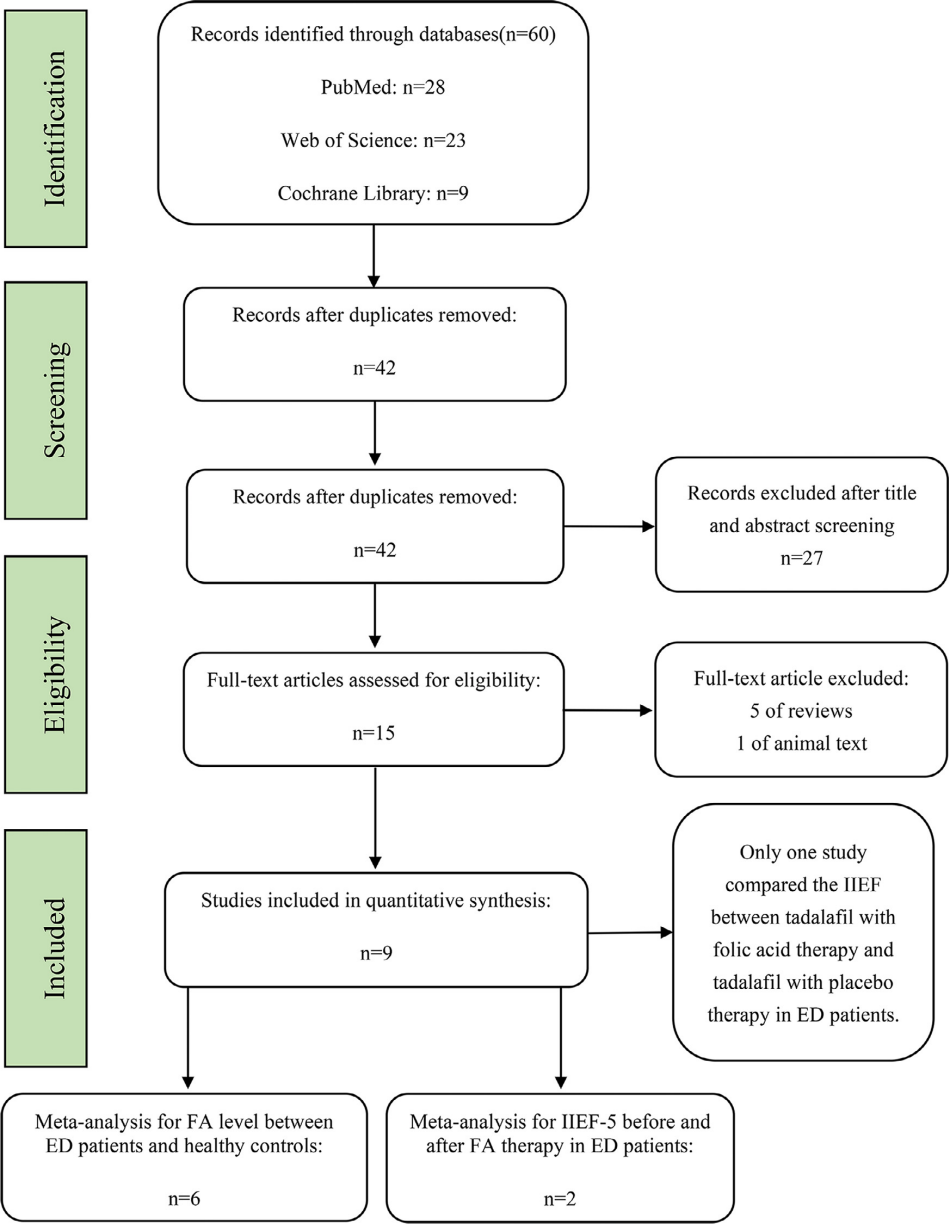
Flowchart of the studies selected for the meta-analysis.

A total of 60 studies were retrieved through the search strategy. We excluded the duplicates through the software of EndNote (version X9.1; Thomson Corporation, Stanford, CT, USA). Then we screened the title and abstracts of the rest including 42 studies, and 27 studies were excluded owing to the unrelated research of our meta-analysis. After reading the full-text of the remained 15 studies carefully, 9 studies were included in our meta-analysis. Among these studies, 6 studies compared the folia acid level between the ED patients and healthy subjects; 2 studies compared the score of the IIEF-5 before and after folic acid therapy in ED patients; Only one study compared the score of the IIEF between tadalafil with folic acid therapy and tadalafil with placebo therapy in ED patients. The process of searching studies was showed in Figure 1.

The Table 1, Table 2, Table 3 showed the characteristics of the included 9 studies. The study year ranged from 2010 to 2020, while only one study was conducted in 2006. There were three studies divided the ED patients into three categories: mild ED,^17^, ^18^, ^19^, ^20^, ^21^ moderate ED^8^, ^9^, ^10^, ^11^, ^12^, ^13^, ^14^, ^15^, ^16^ and severe ED^1^, ^2^, ^3^, ^4^, ^5^, ^6^, ^7^ on the basis of the IIEF-5. The NOS scores of the included studies ranged between 6 and 8, demonstrating the high quality of the included studies. The results of the NOS of the included studies were showed in Table 4.

**Table 1:** Characteristics of included studies comparing the serum FA between ED and healthy controls

				ED patients			Healthy controls		
Author	Year	Country	Definition of ED	Number	Age, year	Folic acid, ng/ml	Number	Age, year	Folic acid, ng/ml
Wenjie Yan	2014	China	IIEF-5<22	42	28.62 ± 4.23	7.61 ± 3.97	30	27.89 ± 3.98	12.23 ± 5.76
M. Karabakan	2016	Turkey	IIEF-5<22	120	—	8.19 ± 3.88	40	56.6 ± 7.5	10.7 ± 4.6
			IIEF-5: 17-21	Mild (40)	54.6 ± 9.2	10.2 ± 4.6			
			IIEF-5: 8-16	Moderate (40)	58.6 ± 8.1	7.1 ± 3.2			
			IIEF-5: 1-7	Severe (40)	53.9 ± 9.5	7.2 ± 3.7			
Massimiliano Sansonea	2017	Italy	IIEF-5<22	31	52.83 ± 11.89	5.11 ± 1.79	31	49.14 ± 13.63	7.9 ± 3.55
Yang Chen	2018	China	IIEF-5<22	688	37.99 ± 10.75	9.56 ± 2.72	693	34.18 ± 8.47	9.89 ± 11.28
Attia Abd Allah Attia	2019	Egypt	IIEF-5<22	60	34.13 ± 6.36	7.1 ± 2.47	30	54.00 ± 9.73	13.36 ± 2.49
			IIEF-5: 17-21	Mild (8)	42.00 ± 3.12	9.91 ± 1.00			
			IIEF-5: 8-16	Moderate (19)	49.05 ± 8.88	7.46 ± 1.09			
			IIEF-5: 1-7	Severe (33)	59.76 ± 6.60	6.18 ± 2.74			
Abhimanyu Gupta	2020	India	IIEF-5<22	41	32.58 ± 8.62	5.29 ± 2.52	36	34.63 ± 10.78	10.8 ± 11.42
			IIEF-5: 17-21	Mild (7)	35.2 ± 5.823	8.28 ± 1.704			
			IIEF-5: 8-16	Moderate (24)	—	5.16 ± 2.23			
			IIEF-5: 1-7	Severe (10)	32.9 ± 8.293	3.5 ± 1.715

**Table 2:** Characteristics of included studies comparing the score of the IIEF-5 before and after folic acid therapy in ED patients

				Before folic acid administration			After folic acid administration		
Author	Year	Follow-up, month	Dose of FA	Number	Age, year	IIEF-5	Number	Age, year	IIEF-5
R. AGOSTINI	2006	3	0.4mg	88	50-70	12 ± 5	88	50-70	20 ± 3
A.R.M. Elshahid	2019	3	0.5mg	50	42.8 ± 6.7	6 ± 0.74	50	42.8 ± 6.7	14 ± 4.44

**Table 3:** Characteristics of included studies comparing the score of the IIEF between tadalafil with folic acid therapy and tadalafil with placebo therapy in ED patients

				Tadalafil (10mg) with folic acid			Tadalafil (10mg) with placebo		
Author	Year	Follow-up, month	Dose of FA	Number	Age, year	IIEF changes	Number	Age, year	IIEF changes
Ali Hamidi Madani	2013	3	5mg	35	55.65 ± 6.22	5.14 ± 3.84	48	57.70 ± 5.98	1.68 ± 0.99

**Table 4:** Quality assessment for all the included studies

	Selection				Comparability		Outcome			
First author (Year)	Case definition adequate	Representativenessof the cases	Selection of controls	Definition of controls	Main factor	Additional factor	Ascertainment of exposure	Same Method of ascertainment for cases and controls	Nonresponse rate	Score
Wenjie Yan (2014)	*	*	*	*	*	*	—	*	—	7/9
M. Karabakan (2016)	*	*	*	*	*	*	—	*	—	7/9
Massimiliano Sansonea (2017)	*	*	*	*	*	—	—	*	—	6/9
Yang Chen (2018)	*	*	*	*	*	*	—	*	—	7/9
Attia Abd Allah Attia (2019)	*	*	*	*	*	*	*	*	—	8/9
Abhimanyu Gupta (2020)	*	*	*	*	*	—	*	*	—	7/9
R. AGOSTINI (2006)	*	—	*	*	*	—	*	*	—	6/9
A.R.M. Elshahid (2019)	*	*	*	*	*	—	*	*	—	7/9
Ali Hamidi Madani (2013)	*	*	*	*	*	*	*	*	—	8/9

*Indicates “fulfilled” or “yes.”

### Folic Acid Level Between ED Patients and Healthy Subjects

**Figure 2:**
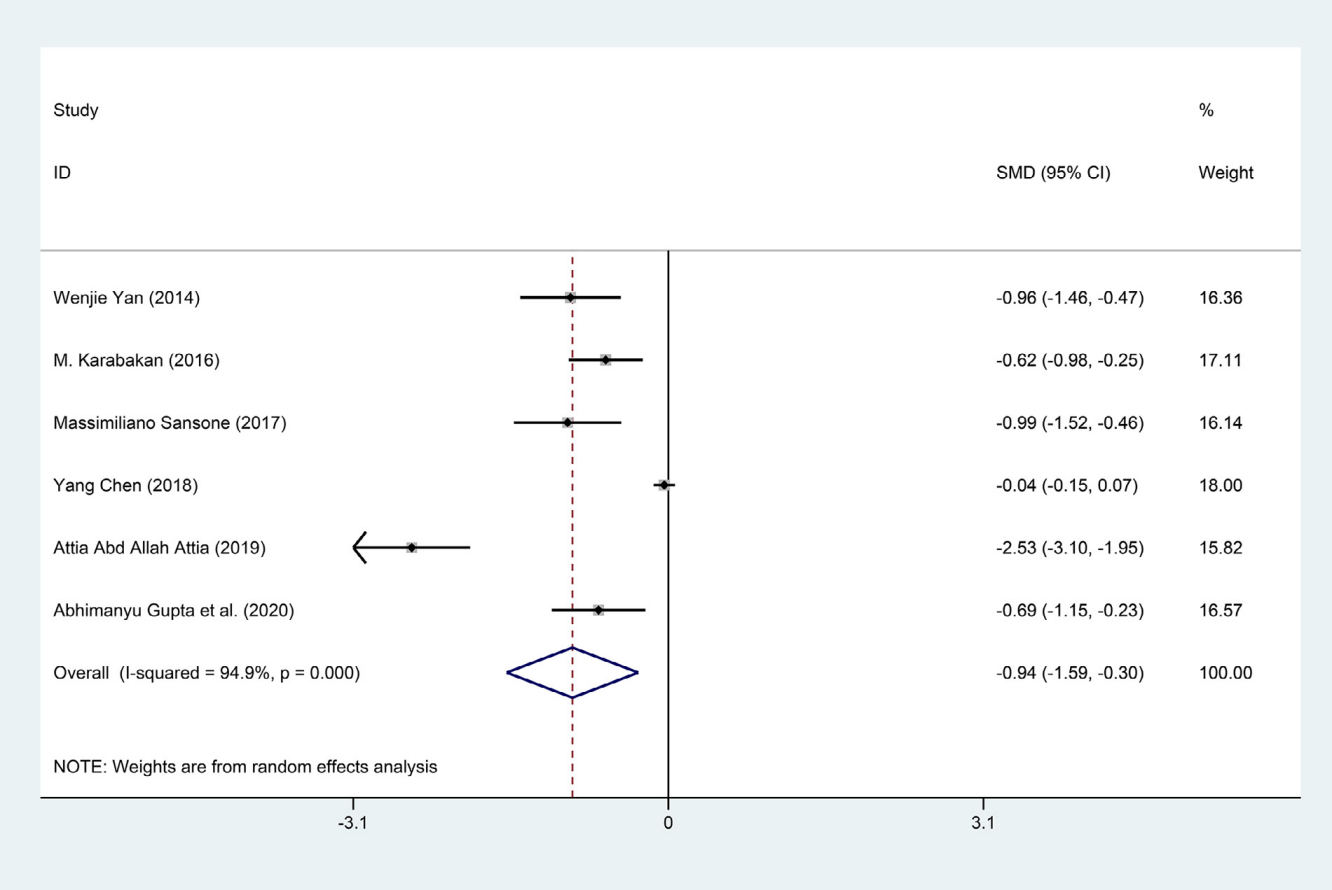
Forest plot for the level of folic acid between ED patients and healthy subjects.

**Figure 3:**
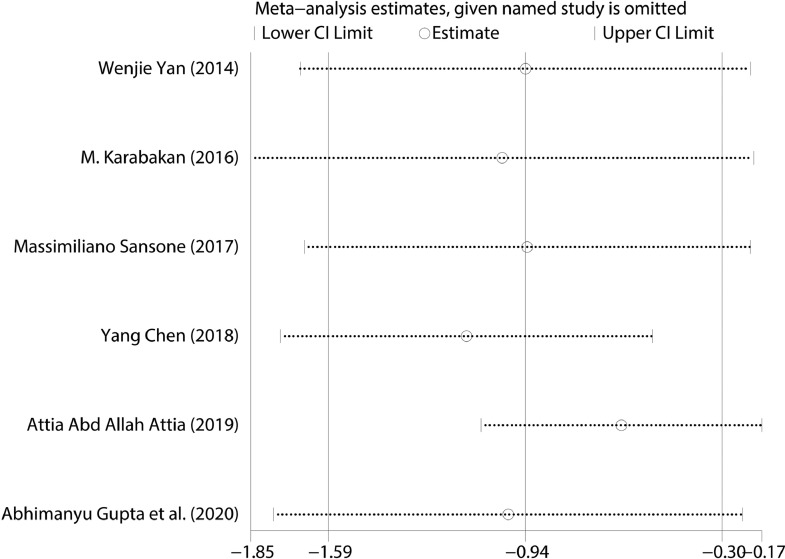
Sensitivity analysis of the selected studies for level of folic acid between ED patients and healthy subjects.

The SMD was calculated to compare the level of folic acid between ED patients and healthy subjects. Six studies including 982 ED patients and 860 healthy subjects compared the folic acid level between the ED patients and healthy subjects.^17^, ^18^, ^19^, ^20^, ^21^, ^22^ The results showed that the level of the folic acid in ED patients had about 0.94 ng/ml lower than that in healthy subjects (SMD (95%CI) = -0.94 (-1.59, -0.30), *P* = .004) with severe heterogeneity existed (I^2^ = 98.15%). Based on the heterogeneity, the SMD with 95% CI was calculated in a random-effect model. The result was exhibited in Figure 2. We performed the sensitivity analysis to assess the stability and reliability of the results of the folic acid level. We omitted one study in each time and the results was consistent. The result of the sensitivity analysis was showed in Figure 3. When using the Begg's test to detect the publication bias, no significant publication bias was found across studies (z = 1.88, *P* = .06).

### Severity of the ED and the Level of the Folic Acid in ED Patients

**Figure 4:**
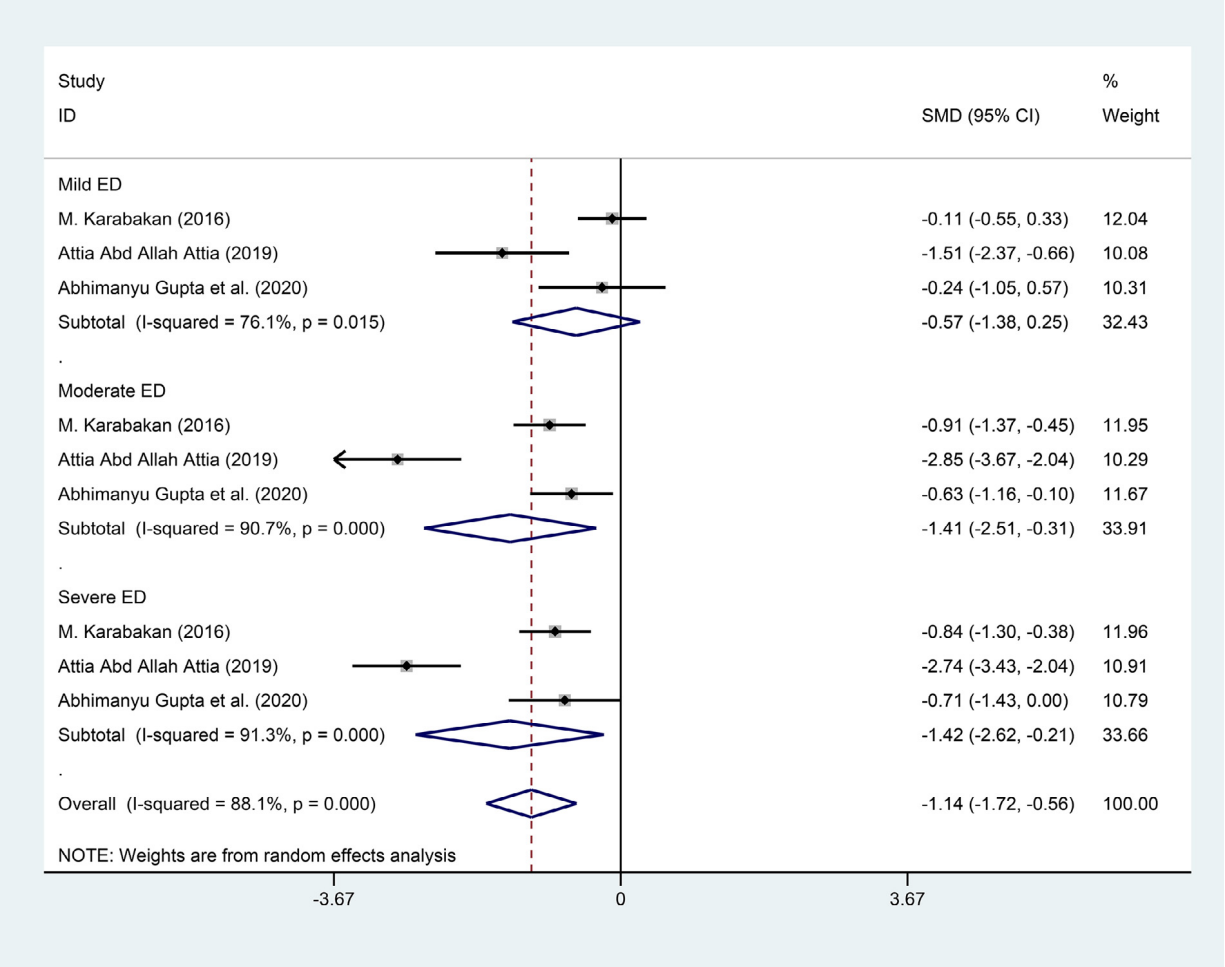
Forest plot for the level of folic acid between Mild ED, Moderate ED, Severe ED and Healthy subjects.

**Figure 5.1:**
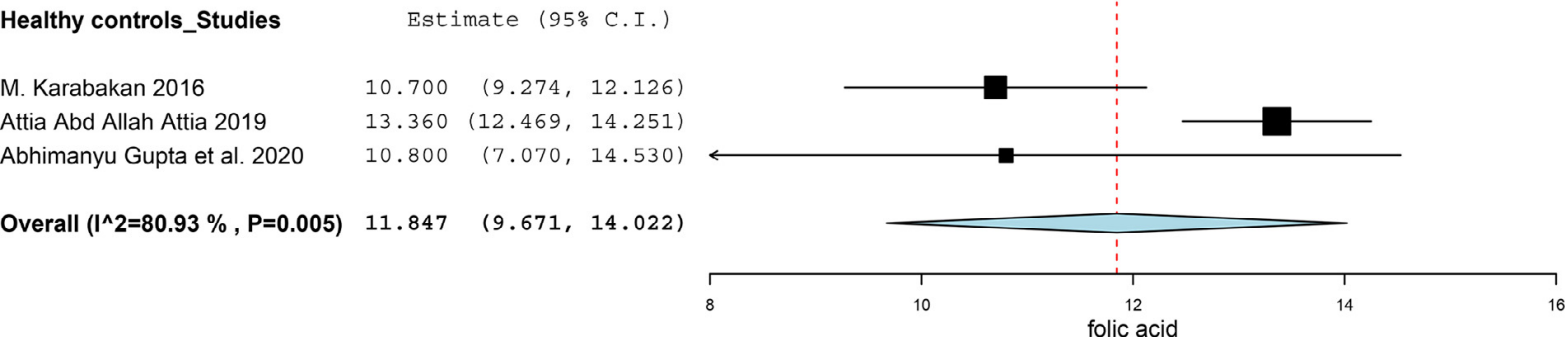
Synthesized results of the level of folic acid in healthy subjects.

**Figure 5.2:**
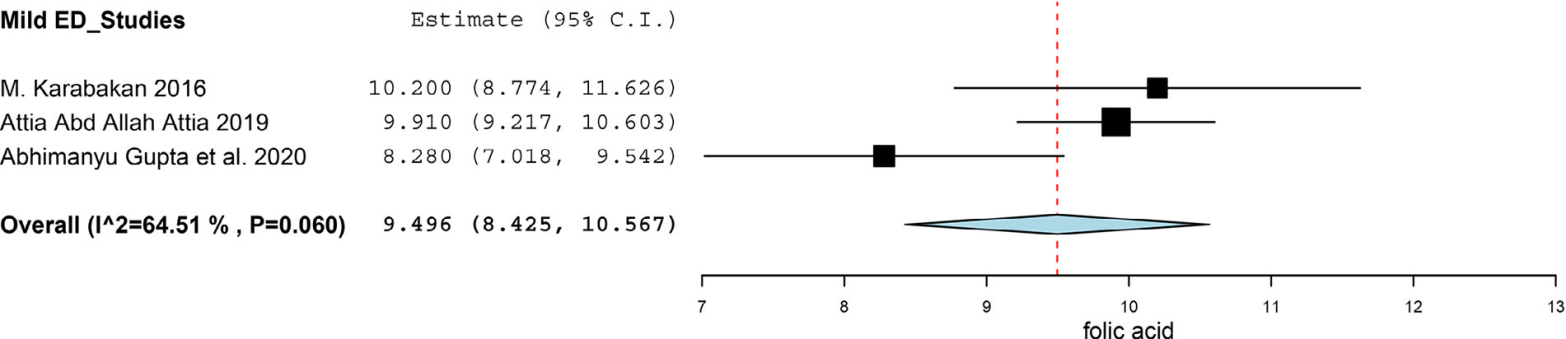
Synthesized results of the level of folic acid in Mild ED patients.

**Figure 5.3:**
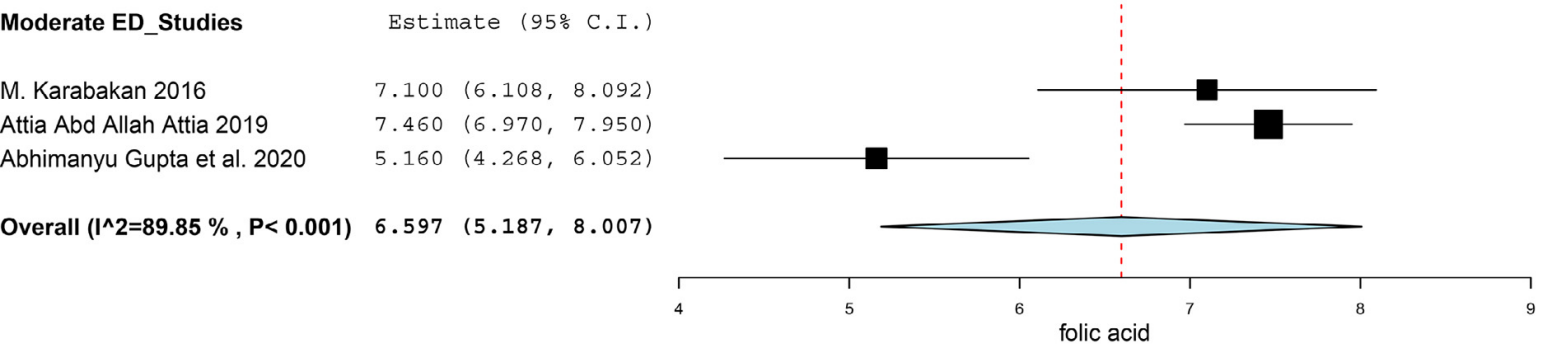
Synthesized results of the level of folic acid in Moderate ED patients.

**Figure 5.4:**
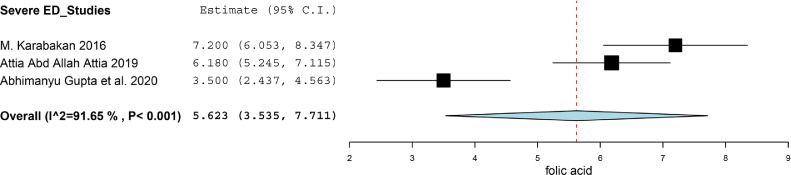
Synthesized results of the level of folic acid in Severe ED patients.

There were three studies divided ED patients into different group on the basis of the score of IIEF-5, and compared the folic acid level between ED patients and healthy subjects.^18^^,^^21^^,^^22^ We calculated the SMD between Mild ED, Moderate ED, Severe ED and Healthy subjects. There was no statistically significant difference between Mild ED and healthy subjects (SMD (95%CI) = -0.57 (-1.38, 0.25), *P* = .172, I^2^ = 76.1%). As for the Moderate ED patients, they had about 1.41 ng/ml lower than that in healthy subjects (SMD (95%CI) = -1.41 (-2.51, -0.31), *P* = .012). The Severe ED patients had about 1.42 ng/ml lower than that in healthy subjects (SMD (95%CI) = -1.42 (-2.62, -0.21), *P* = .021). The result was showed in Figure 4. We synthesized the results of the ED patients and healthy subjects respectively to find the correlation between the severity of the ED and the level of folic acid. The synthesis of the results was conducted by using the software of Open Meta-Analyst. The level of folic acid in healthy subjects, Mild ED patients, Moderate ED patients and Severe ED patients was 11.847 (95%CI = 9.671, 14.022), 9.496 (95%CI = 8.425, 10.567), 6.597 (95%CI = 5.187, 8.007) and 5.623 (95%CI = 3.535, 7.711) respectively. We exhibited the result in Figures 5.1-Figure 5.1, Figure 5.2, Figure 5.3, Figure 5.4.

### Changes of the IIEF-5 With the Folic Acid Administration in ED Patients

**Figure 6:**
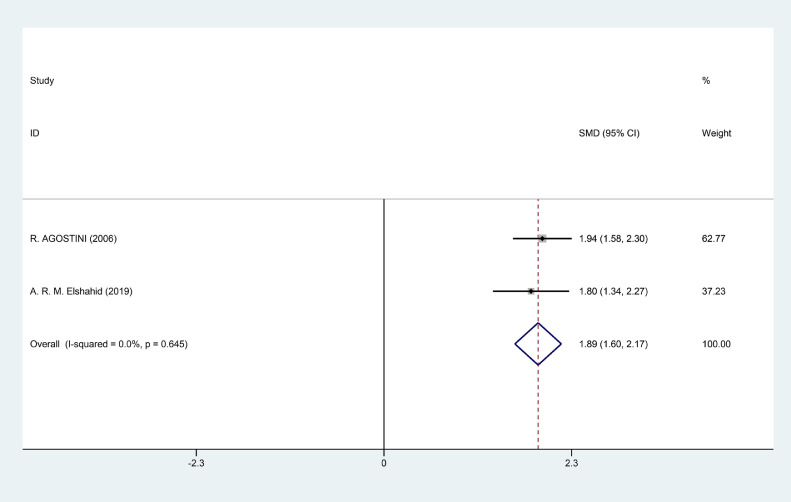
Forest plot for the IIEF-5 before and after folic acid administration.

**Figure 7:**
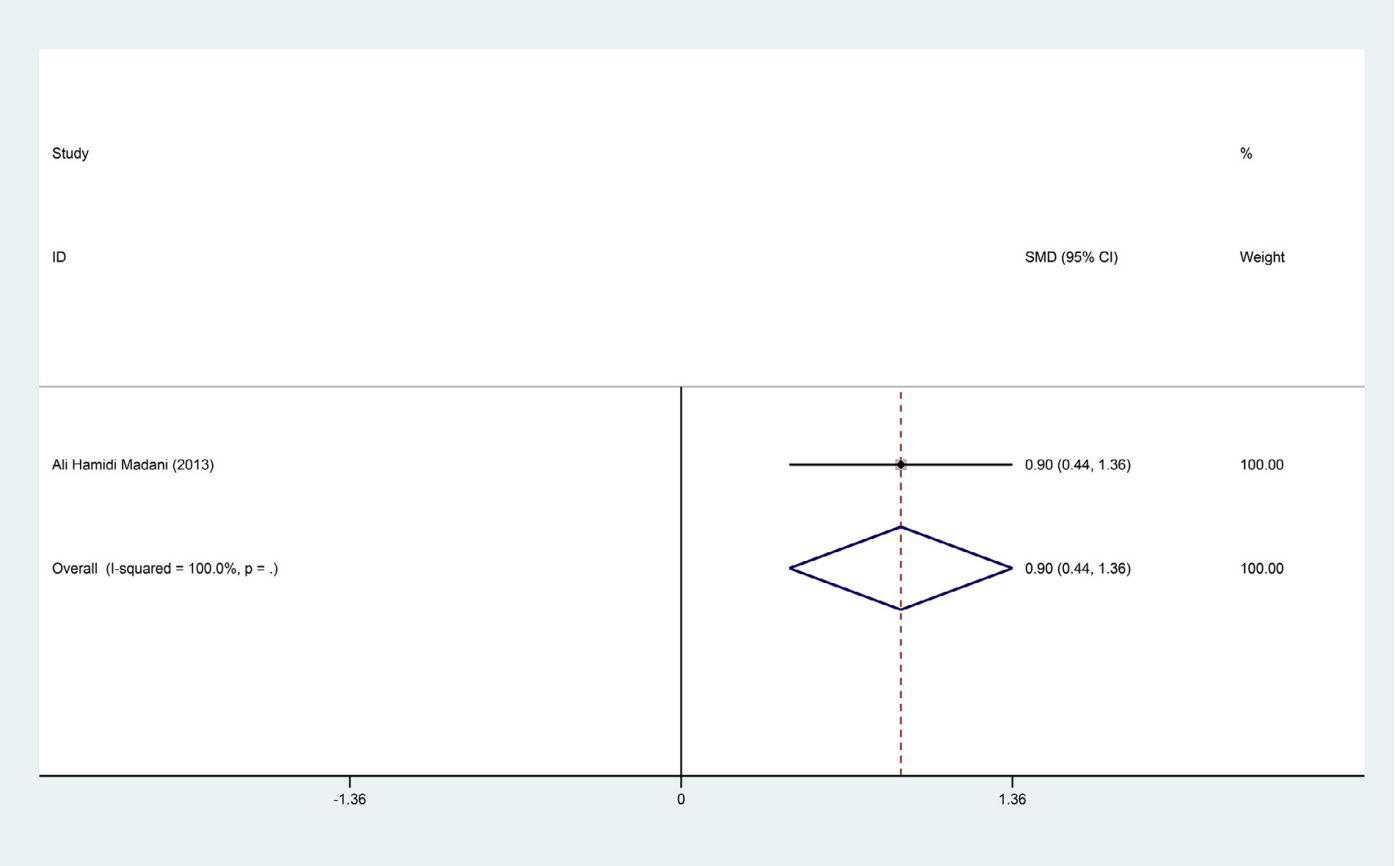
Forest plot for the IIEF between folic acid combining tadalafil and placebo combining tadalafil.

Three studies were involved in comparing the IIEF-5 before and after folic acid administration. The changes of the IIEF-5 after the folic acid administration only were reported in two articles.^23^^,^^24^ Fixed-effect model was used to calculate the SMD owing to the no significant heterogeneity (I^2^ = 0.00%, *P* = .645). The SMD was 1.89 with the 95%CI (1.60, 2.17). Only one study was included in comparing the IIEF between folic acid combining the tadalafil and placebo combining the tadalafil.^25^ The pooled result showed that folic acid combining tadalafil had about 0.90 (95%CI = 0.44, 1.36) higher than that in placebo combining tadalafil administration of the IIEF scores. The results were showed in Figures 6 and 7 respectively. Notably, the statistical differences of IIEF-5 or IIEF may be driven by large sample size. It didn't meet the bar for clinical significance to ED therapy.

## DISCUSSION

This comprehensive study confirmed that serum FA was correlated with ED and the serum FA decreased significantly with the severity of ED. Moreover, our study demonstrated that FA administration could improve the score of IIEF-5 in ED patients. When comparing the FA between ED patients and healthy subjects, we enrolled 6 studies with 982 ED patients and 860 healthy subjects, which all were regarded as high-quality literature using NOS quality scores. It is regretful that we only enrolled 3 studies when comparing the IIEF-5, or IIEF changes before and after FA administration. But the conclusions were consistent to the relationship between FA and ED. We conducted the first meta-analysis to date to demonstrate the role of FA in the pathology of ED.

The EnD was correlated with the pathogenesis of ED significantly. So, a number of studies concentrated on exploring the laboratory factors in the pathogenesis of ED, which including serum testosterone level, serum vitamin D level, and platelet indices, resulting in ED owing to the destruction of the endothelial function.^26^, ^27^, ^28^ Recently, several studies have found that the FA supplementation could improve the endothelial function in patients with DM and hypertension.^29^^,^^30^ A new question was raised by the researchers that whether deficiency of FA could lead to the occurrence of ED. Several prospective studies were conducted to compare the FA level between ED patients and healthy subjects and compare the FA level of different severity of ED divided by the score of IIEF-5. Wen‑Jie Yan conducted the first study to investigate the relationship between FA and ED. They demonstrated that ED patients had lower serum FA concentrations (7.61 ± 3.97 ng/ml) than that in healthy subjects (12.23 ± 5.76 ng/ml).^17^ They also found the positive correlation between the FA level and IIEF-5 scores (r = 0.589). On the contrary, another study didn't find any association between FA and ED, of course the FA level and the severity of ED.^20^ Actually, this study was the first and the only one to conclude the no significant correlation between FA and ED. When synthesizing all the data published with large study population, we concluded the positive correlation between FA and ED, also the negative correlation between FA level and severity of ED.

FA had important role in the metabolism of NO and homocysteine (Hcys).^12^^,^^31^ The FA was related to the metabolism of Hcys through remethylating it to methionine.^17^ However, hyperhomocysteine has been found to be a novel risk factor for ED owing to its inhibitory effect on endothelium-dependent NO formation.^32^ So many studies concluded that FA deficiency resulted in the occurrence of ED by decreasing the metabolism of Hcys, leading to the hyperhomocysteine. This phenomenon seemed to explain the relationship between FA and ED. However, another well-designed study raised a different perspective. No correlation between FA and hyperhomocysteine was found by their study in ED group (r = -0.247, *P* = .197) and control group (r = -0.163, *P* = .372) respectively(19). The FA should be an independent risk factor for ED.

Another way involved in the causation of ED by FA was the tetrahydrobiopterin (BH4), essential for the synthesis of NO.^13^ FA was responsible for the deficiency of BH4. Chemical studies concluded that BH4 was the co-factor of the eNOS. The structural integrity of the Endothelial eNOS was necessary for the formation of NO. So, FA deficiency was responsible for BH4 deficiency, which was responsible for the uncoupling of the eNOS. These pathways could explain our conclusion that FA deficiency was responsible for the causation of ED and FA level decreased following the ED severity increased.

In order to further investigate the influence of FA administration in IIEF-5 score in ED patients, several studies were conducted to compare the IIEF-5 changes in ED patients after the FA administration. They all concluded a highly significant increase of IIEF-5 after FA administration (20 vs 12, 15.5 vs 6.4 respectively).^23^^,^^24^ After pooling the data, the conclusion was consistent. Interestingly, one study of the two further compared the Hcys level before and after FA administration (0.36 vs 0.15, *P* < .001). the group thought that FA administration decreased the Hcys level, improved the NO production, consistent with the former views. But they didn't measure the FA level in ED patients and compared it with healthy subjects. Considering the inhibitory effect of FA in uncoupling the eNOS, several studies suggested that FA improved the endothelial function independent of its effect in lowering the Hcys.^29^^,^^33^ But the positive role of FA in the treatment of ED couldn't be verified from our meta-analysis. First, only 3 studies were included in our meta-analysis. Second, the improvement of IIEF-5 or IIEF before and after folic acid administration could be driven by large sample size. It didn't meet the bar for clinical significance to ED therapy. So future researches are needed to explore the treatment efficacy of FA for ED patients.

Ali Hamidi Madani et al conducted a randomized controlled trial to investigate that if the combination with FA plus tadalafil could improve the IIEF scores more efficient than placebo plus tadalafil in ED patients with type 2 DM. After three-month follow up, the group with FA plus tadalafil accomplished larger changes of IIEF than that in group B with placebo plus tadalafil (5.14 ± 3.84 vs 1.68 ± 0.99, *P* = .001).^25^ This study further confirmed the efficacy of FA in therapy of ED, even in ED with DM. The etiology of ED in DM patients is multifactorial. However, the deficiency of NO was the prominent factor of ED patients with DM.^34^ So, FA could restore endothelial function by increasing the NO availability. Actually, another supplement, arginine could treat ED with the same pathway.^35^ It played an important role in the NO-producing pathway. Although the first-line treatment for ED was phosphodiesterase (PDE) type 5 enzymes. It is potential to prescribe tadalafil plus FA for ED patients, without severe adverse effects.

Among included studies, Attia et al conducted a prospective study to compare the FA level between ED patients and healthy controls. Worthwhile, the control group in their study contained 30 healthy subjects with age 54.00 ± 9.73 elder than that in ED group. But it couldn't influence the conclusion of our meta-analysis. Abhimanyu et al have verified no correlation between FA level and age (r = 0.138, *P* = .389). Another study conducted by Massimiliano et al also demonstrated that FA level was unrelated to the age (r = 0.225. *P* = .290). So the difference of age between ED patients and healthy subjects couldn't influence the results of our study.

When an ED patient referred to an andrologist, he would be checked for serum testosterone, sugar, and lipid for looking for reversible risk factors. Our meta-analysis could remind the andrologist that low FA level would be a risk factor for ED patients, worthy for checking to find the etiology of ED. Moreover, our conclusion could also supply a novel therapy choice for ED besides PDE5i only.

Our meta-analysis has some limitations. We only included three studies when evaluating the FA treatment efficacy for ED. But we are the first one to explore the role of FA in the pathology and therapy of ED. We included enough studies to verify the association between FA and ED. More well-designed randomized controlled studies were needed for assessing the treatment efficacy of FA for ED. Secondly, our study had higher heterogeneity. In order to solve the heterogeneity of enrolled studies, subgroup analysis and sensitivity analysis were conducted. Partial heterogeneity could be attribute to subgroup. Other reason responsible for the heterogeneity included sample size, patients age, duration of ED. Last but not least, the diagnosis of ED was based on the questionnaire of IIEF-5 instead of clinical investigation such as RigiScan, penile Doppler ultrasonography. Several strengths of our meta-analysis make our study worthwhile to be conducted. First, our study was the first meta-analysis to verify the relationship between FA and ED. Second, we included larger sample to provide more convincing evidence than each original research with small sample size. But, more prospective studies are needed in the future to make up the limitations of our review studies.

## CONCLUSION

In conclusion, our meta-analysis confirmed that serum FA level was associated with ED tightly. In a word, serum FA deficiency could be an independent risk factor for ED. And the serum FA level decreased significantly with the severity of ED. Even though, our study couldn't supply sufficient evidence to demonstrate the positive effect of folic acid for managing ED. But, the potential positive effects of FA in treatment of patients with ED worth more cohort study in the future.

## STATEMENT OF AUTHORSHIP

Yuyang Zhang: Conceptualization, Methodology, Software, Formal Analysis, Data Curation, Writing – Original Draft, Writing – Review & Editing, Project Administration; Wei Zhang: Methodology, Software, Formal Analysis, Writing – Review & Editing; Yutian Dai: Software, Writing – Review & Editing, Project Administration; Hui Jiang: Conceptualization, Writing – Review & Editing, Project Administration; Xiansheng Zhang: Conceptualization, Methodology, Formal Analysis, Data Curation, Writing – Original Draft, Writing – Review & Editing, Project Administration, Funding Acquisition.
